# Leading by the nodes: a survey of film industry network analysis and datasets

**DOI:** 10.1007/s41109-024-00673-9

**Published:** 2024-12-18

**Authors:** Aresh Dadlani, Vi Vo, Ayushi Khemka, Sophie Talalay Harvey, Aigul Kantoro Kyzy, Pete Jones, Deb Verhoeven

**Affiliations:** 1 Department of Mathematics and Computing, Mount Royal University, Calgary, Alberta Canada; 2 Faculty of Arts, University of Alberta, Edmonton, Alberta Canada

**Keywords:** Social network analysis, Film industry, Node classification, Collaboration networks, Literature survey

## Abstract

This paper presents a comprehensive survey of network analysis research on the film industry, aiming to evaluate its emergence as a field of study and identify potential areas for further research. Many foundational network studies made use of the abundant data from the Internet Movie Database (IMDb) to test network methodologies. This survey focuses more specifically on examining research that employs network analysis to evaluate the film industry itself, revealing the social and business relationships involved in film production, distribution, and consumption. The paper adopts a classification approach based on node type and summarises the key contributions in relation to each. The review provides insights into the structure and interconnectedness of the field, highlighting clusters of debates and shedding light on the areas in need of further theoretical and methodological development. In addition, this survey contributes to understanding film industry network analysis and informs researchers interested in network methods within the film industry and related cultural sectors.

## Introduction

In this paper, we present a survey of published research applying network analysis methods to the study of the film industry. The film industry has long been a rich source of information for network analysis. Use of the Internet Movie Database (IMDb) is prevalent in foundational network studies that require exemplary real-world applications for network theory (Barabási and Albert 1999; Ahmed et al. 2007; Madduri et al. 2009; Fatemi and Tokarchuk 2012; Gallos et al. 2013). DiMaggio (2011) has identified the ready availability of film industry data via the IMDb API as a key determinant in a range of defining studies. The memorable Six Degrees of Kevin Bacon game (Fass et al. 1996) embedded the idea of the film industry as a collaboration network in the popular imagination. For academic studies, it was Watts (1999) that used this same dataset to establish a long line of work on small-world collaboration networks based on film data as a use case (Amaral et al. 2000; Rozenfeld et al. 2010; Gallos et al. 2013). Given the critical importance of film industry data to the theoretical development of network science, we were interested to switch perspective to understand the ways in which network science has developed a view of the film industry as an inherently collaborative sector. In this article, we present the first systematic survey of this area of research, in order to guide network scientists interested in analysing the film industry on how they might usefully approach this sector given what has already been done.

For this survey we are interested in papers that do more than simply use film industry data to test a network methodology or theory. Our focus is on papers that use network analysis to provide an evaluation of the film industry itself. The film industry is interwoven with manifold connections, involving filmmakers, actors, production companies, distributors, venues, and cinemagoers, each contributing to a rich tapestry of inter-relationships. This intricately structured ecosystem is a compelling domain for network analysis, which holds the potential to reveal crucial insights into patterns of industry collaboration, influence, and resource allocation. Network analysis of the film industry seems especially relevant for making visible the social and commercial relationships that inform the production, distribution, and consumption of the cinema (Verhoeven et al. 2020), and could be useful within the industry itself, for optimizing marketing strategies, distribution channels, and ultimately enhancing a film’s box-office performance.

Our overarching goal in surveying this literature is twofold: firstly, to establish what has been undertaken in this area, and secondly, to evaluate how scholarship focussed on film industry networks has evolved as an emerging field of study. In short, our survey of the literature reveals several broad trends and patterns. Initial film network research for example is primarily focused on individuals, with recent papers adopting an expanded scope including production companies and geographic locations. Additionally, we observe a disproportionate emphasis on the US film industry in the foundational literature with this bias only amplified by repeated engagement and cross-citation in subsequent papers. We outline a more detailed account of these observations at the conclusion of this essay.

Our aim in providing these insights is to assist scholars interested in utilizing network methods to explore the film industry, as well as other cultural industries, by demonstrating where existing scholarship has clustered into emergent debates and approaches, and where new research might be directed. In particular, we highlight the research lines that have advanced the least, and identify gaps in the existing literature that future network analyses of the film industry could address.

## Scope and analytical strategy

The scope of our survey includes any scholarly work using network analytic methods to study film, with some defined exclusions. First, to ensure that we were able to comprehensively read and review the papers, we restricted our review to English language publications. Second, we did not consider theses for our review. Third, we excluded papers which were not substantively interested in film but used a film-related dataset purely to illustrate a methodological development in network analysis. Fourth, we excluded papers using network approaches to represent film narratives as networks of interactions or co-occurrences between characters, as there already exists a comprehensive survey of the character networks literature which covers this type of study (see Labatut and Bost 2019). Finally, we excluded network-oriented papers which do not attempt to represent the networks they describe as data. This helped us keep a check on the many varied ways in which ideas of “networks” are used discursively in different disciplines, many of which do not align with the type of network analysis we survey in this review. As a consequence of this final criterion, we do not include papers here such as Coe (2000) which provide rich and valuable qualitative evidence of the importance of personal networks in the economic geography of film production, but do not contain any data-driven network analysis per se.

We used a variety of search systems and keywords (“imdb”, “movie”, “film industry” in combination with “networks”, “SNA”, “network analysis”, “small world”, “centrality”) to identify papers across a range of platforms (Google Scholar, ResearchGate, Academia.edu, university library catalogues and journal aggregator databases). Once we had compiled an initial list of papers returned from keyword searches, we chained the references in these papers to find further papers. Additionally, we examined each author’s publication list to ensure no relevant papers were overlooked. New papers appeared as we were searching, so we set a publication expiry date for the end of the calendar year 2022.

**Fig. 1:**
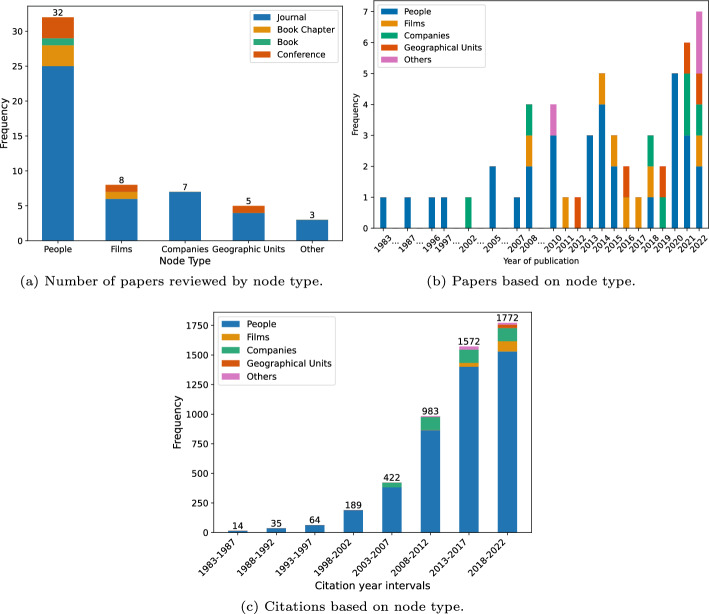
Frequency distributions of **a** the number of papers reviewed by node type, **b** the papers based on node type from years 1983 to 2022, and **c** the citations per each node category over the years. Note that the numbers by node type do not sum to 51 as papers may analyze more than one type of node

Our comprehensive search efforts yielded 51 papers for review. During the review process, we documented information about the type(s) of nodes represented in the network and the publication format (e.g. journal articles, book chapters). These are summarized in Fig. 1a. After the search was complete, we recorded the total number of citations of each paper according to Google Scholar, providing an additional metric for assessing the impact and significance of the reviewed literature. Figure 1b showcases the frequency of publications over time based on the node type. Notably, there is an escalating interest in network heterogeneity in recent years, underscoring that network research of film industries is ongoing and continuing to expand. The citation distribution in Fig. 1c clearly shows a growing focus on networks of individuals over the examined period, while research concerning networks comprised of geographic units remains in its nascent phase.

## Survey of the literature

Survey papers typically adhere to the established structure of their respective fields. However, the literature we review spans multiple disciplines, including film industry studies, business studies, cultural policy studies, sociology, and computational sciences. These disciplines and research fields are structured very differently which complicates the task of conducting a coherent survey. One solution would be to categorize the papers by field or discipline. However, discerning the appropriate disciplinary classification for certain papers is not a straightforward process. Another conventional approach would be to distinguish papers based on different network methods. Our emphasis however was on social network papers evaluating the film industry rather than studies simply using film industry data to explicate or illustrate a network method. Excluding purely method-driven papers made this organisational principle less useful.

To address these challenges, we devised a novel solution involving the classification of papers based on node types. Through systematic examination, we found that scholarship exploring networks of individuals involved in the filmmaking process is often substantively and methodologically different from research focused on connections between films, organizations, events, or geographical units. Although a small number of research papers investigate distinct networks with different node types, to our knowledge there has been no significant study to date that considers the interdependencies among multiple types of nodes within film networks.

### Node type: people

#### Careers and networks

In a groundbreaking work on the careers of screen composers in Hollywood, Faulkner (1983) demonstrates that the industry is dominated by a select group of highly productive and prominent individuals who hold significant control over resources and career opportunities. Through the use of statistical analysis and personal interviews with both established and peripheral composers, Faulkner traces a network linking filmmakers (employers) and composers (employees) to reveal patterns of work distribution and labor division. Building on this study, Faulkner and Anderson (1987) uncover connections between independent contractors in the film industry based on their co-working and contracting patterns. Using data from 2,430 films produced between 1965 and 1980, they identify trends in the repeated connections among participants with similar levels of cumulative productivity, measured by earnings, Oscars film credits. They note that film producers tend to hire directors with a higher earning history, and lower-earning producers work with lower-earning directors. Similarly, directors with a successful track record of high-grossing films contract actors and cinematographers with similar histories. For Faulkner and Anderson these patterns demonstrate that Hollywood’s collaboration network is based on reputation and that career navigation is driven by interpersonal signals and connections.

In another paper on the role of social networks in project-based careers, Jones (1996) addresses the questions: How are careers and work structured in the dynamic and decentralized film industry of Hollywood? What expertise and knowledge is vital for career success when participation is renegotiated for each project? How do careers and projects align to maintain a networked organization? Are boundaryless (project-based) careers primarily based on temporary transactions, or repeated interactions that foster learning and innovation? The author analyzes the project network in the US film industry (1977-79) using *k*-core analysis. They show that to achieve success, individuals aim to acquire new technical skills and/or form connections to become part of more production teams. Similarly, Jones and Walsh (1997) explore how independent contractors navigate the US film industry and how their position within the core or periphery of the network impacts their careers. The authors also examine labor market stratification and segmentation in the industry between 1977-79. Their study was informed by in-depth interviews with five industry professionals, along with publication records and film credits. Findings highlight fluid entry and exit points for contractors and companies. The authors perform a *k*-core analysis which demonstrates that major studio-affiliated contractors in the central core have better opportunities for high-paying and challenging film projects.

Drawing on this earlier work, Delmestri et al. (2005) explore the importance of reputation and collaboration networks on movie project success. They hypothesize that economic reputation (past box office success) affects commercial achievement, while artistic reputation (favorable critical reviews of past films) influences artistic merit. For network effects, they turn to the organizational literature to hypothesize that strong vertical ties (dyadic ties between the director and the distributor and producer) will be positively associated with economic success, while strong horizontal ties (dyadic ties between the director and scriptwriter, director of photography and lead actor) will be negatively associated with artistic merit. Their methodology combines interviews with 14 professionals in the Italian film industry and quantitative data on Italian films from 1990 to 1998. The authors find support for each of their hypotheses, though the negative effect of strong horizontal ties on artistic merit is only marginally significant.

#### Networks, stability and innovation

Ferriani et al. (2005) present the film industry as an example of temporary project firms that maintain stability despite their transitory nature. They study enduring interpersonal relationships within the industry that persist after the completion of individual projects and the disbandment of project firms themselves. Collaborations between various participants in the industry (such as directors, sound mixers, and producers) reveal underlying factors driving organizational knowledge and practice. The paper uses IMDb (cross-checking against the Alan Goble Film Index) to locate and analyze movies distributed in the United States by the seven major studios and the two largest independent subsidiaries (Miramax and New Line) between 1995 and 1999. They find that the repeated collaborations in the industry give rise to latent networks, which counter the lack of permanent organizational structures. The authors acknowledge they cannot definitively determine whether repeated collaborations occur due to friendship and consider their findings to be suggestive rather than conclusive.

Perretti and Negro (2007) analyze film genre innovation in the film industry by assessing the introduction of new genres and their combinations. They hypothesize that a higher proportion of newcomers and a higher proportion of new combinations of both newcomers and old-timers would be positively associated with innovation. The authors tested these hypotheses on the U.S. movie industry by analyzing 6,446 titles produced by major Hollywood studios from 1929 to 1958. The method involved classifying films as single-genre or multi-genre and classifying production teams as combinations of newcomers, newcomers and old-timers, or old-timers. They concluded that the rate of innovation was indeed increased by the introduction of newcomers, and that innovation was further increased when teams combined newcomers with old-timers. The study also found that executive-level individuals (managers) had little impact on innovation.

In a particularly influential paper, Cattani and Ferriani (2008) introduce a new perspective on individual-level creativity in which they propose that individuals positioned between the core and periphery of their social network are more likely to achieve creative outcomes. This perspective can also apply to teams, where individuals in a team that have members from both ends of the core/periphery spectrum have an advantage. The study focuses on Hollywood between 1992 and 2003 and uses data of all core crew members who worked in movies distributed by the eight major studios. The first hypothesis is that individual creative performance has an inverse U-shaped relationship with their coreness, implying that intermediate positions yield the highest creative performance. The second hypothesis is that the relationship between individual creative performance and team coreness is also an inverted U-shape, with optimal creative performance achieved by individuals in teams with a moderate level of coreness. The study concludes that individuals bridging core and periphery boundaries enjoy enhanced creative performance, and moderate levels of coreness are beneficial. Teams with a balanced core-periphery mix experience similar benefits. The authors underscore the industry-specific context and acknowledge that creativity is outcome rather than process-focused in this study.

#### Networks and success prediction

Krauss et al. (2008) also focus on understanding film performance outcomes in their investigation of whether opinions expressed by IMDb forum users can be used to predict Academy Award nominations and box office success of unreleased films. The first model used the “Oscar Buzz” subforum of IMDb and determined the likelihood of a film receiving an Academy Award nomination by computing three factors: the Intensity Index, the Positivity Index, and the Time Noise Factor. The second model analyzed 20 unreleased films and created a “Buzz Mode” from the Intensity, Positivity, and Trendsetter Indexes. The Trendsetter Index was calculated by identifying subforum users with the highest betweenness centrality in the “Previews and Review” subforum and normalizing their number on a scale of 0 to 1. Results affirm both hypotheses: positive “Oscar Buzz” discussions are related to Oscar nominations, and positive discussion on the “Previews and Reviews” subforum correlated with higher box office revenue.

Box-office prediction is also the focus of a study by Karpov and Marakulin (2022) which aims to predict a movie’s success by utilizing its position within the community structure of the international film industries alongside conventional film-level variables such as language, country, rating and genre. Using IMDb data, the authors construct a network connecting actors, casting directors, talent agents, and directors based on co-affiliations with movies. They demonstrate that incorporating additional information derived from this network can improve the accuracy of predicting a movie’s success by up to $6\%$, depending on the classification method. They also find that the importance of a casting director is greater than that of actors in terms of predicting a movie’s success, which offers the opportunity to predict a movie’s success at an early stage, even before actors are approved for roles.

An earlier analysis by Meiseberg and Ehrmann (2013) retrospectively examined the relationship between a film’s creative team composition and its success in domestic and international markets. They develop hypotheses about team diversity in terms of personal attributes, film locations, and social ties, and test them using ordinary least squares (OLS) regression models. They found that diverse nationalities and star status, as well as multiple film locations, are the strongest predictors of export success. For domestic success, positive factors include diversity in the age and tenure of team members, and the number of connections in the people-film network. Gender diversity however, was negatively associated with domestic success. They conclude that the right mix of team attributes, experience, and backgrounds influences success, and reducing cultural specificity enhances international market success.

Viana et al. (2014) also study film success in relation to the historical development of team composition in the film industry by examining topological (small world coefficient, betweenness, closeness, local clustering coefficient) and non-topological (previous experience) properties of directors, producers, and writers. Using IMDb they identified a set of 3,006 films from the late 1800 s to 2013 that had received a minimum of 25,000 user votes and including films from the IMDb top 250 list. They then analyze the correlation between these properties and film success, measured by gross income and ratings. They discover that non-topological properties have a stronger relationship with film success, while topological properties have a weaker correlation. Additionally, they find that teams with less experienced members tend to perform better, indicating that bringing in new talent can improve a team’s success.

Rather than define success in terms of box-office income, Rossman et al. (2010) use data on Academy Award acting nominations to examine the relationship between artistic achievement and collaboration in the film industry. The authors analyzed actors in films released between 1936 and 2005, while controlling for factors such as the actor’s personal history and basic film traits. Data from the Academy of Motion Pictures Arts and Sciences (AMPAS) and the Internet Movie Database (IMDb) was used to create a dataset of the top 10 credited actors. Only films eligible for Academy Awards from 1936 to 2005 were analyzed, a total of 16,392 films featuring 147,908 performances by 37,183 actors. Of these, only 1,326 performances received Oscar nominations and 279 wins, which is less than $1\%$ of the total performances. Using statistical analysis and logistic regression, the study found that actor’s status, as measured by their asymmetric centrality in the network of screen credits, is a strong predictor of their chances of receiving an Oscar nomination. Additionally, actors are more likely to receive an award when they work with elite collaborators.

Ebbers and Wijnberg (2010) aim to understand the impact of performance-based reputation on the development of alliance networks in the Dutch film industry over time. The authors use stochastic actor-oriented models to examine the impact of an actor’s reputation, which is based on their previous performance, on the formation of alliances, while controlling for constant actor attributes and network position. They differentiate between individual reputation and composite reputation, considering past organizational involvement and partner reputations. Results show that an actor’s reputation (with a focus solely on artistic reputation derived from reviews) and their proximity in past alliance networks are strong indicators of alliance formation. The study suggests that actors with similar reputations are more likely to form alliances with each other. The authors performed additional analysis of the impact of commercial reputation, based on a return-on-investment measure using box-office and budget data, but no significant effects were found.

#### Historical studies

Two studies use data related to the Hollywood “blacklist” era in which alleged communist sympathizers were investigated by the House Un-American Activity Committee (HUAC). Pontikes et al. (2010) study the role of network diffusion processes for stigmatization. They propose hypotheses about the effect of associating with a later-blacklisted artist on work opportunities. Testing with logit regression models, they found that mere association with a blacklisted artist reduces the odds of working in the next year, and that only one instance of mere association was enough for this negative effect to apply. The effect is stronger for actors collaborating with blacklisted writers, suggesting stigma spreads through diverse connections. They also observed that high-profile actors are not immune to the effects of stigma. In a related study, Negro and Goodman (2015) explore whether niche overlap (two social actors in the Hollywood labour market with similar professional and demographic features) is connected to the act of naming somebody in the HUAC trials. Their hypothesis is that niche overlap is positively related to the likelihood of discrediting. They use data from published reports of the public hearings to identify potential discreditors and their actions in a binary discrediting network. The set of people is limited to actors, directors, writers, and producers involved in commercial feature-length films up to the conclusion of the hearings. They use a naming network, a collaboration network, and combinations of these to define relational patterns associated with the act of discrediting. Employing a multi-level logit model with crossed random effects, and while controlling for various factors including prior collaborations, they found compelling evidence linking niche overlap to an increased likelihood of discrediting.

#### Gatekeeping

Network analysis has proved especially valuable to several authors exploring the importance of gatekeeping as both a commentative and as an employment practice in the film industry. Cattani and Ferriani (2013) test the hypothesis that industry peers who act as evaluators tend to reinforce dominant social beliefs and norms, and are more likely to acknowledge core members over peripheral network members. Using Hollywood data from 1992 to 2004 they constructed film collaboration networks and analyzed the bipartite affiliation networks by creating an individual-by-movie matrix. The authors also created an “accumulative relational profile” by using moving windows of three-year intervals. Their findings suggest that social relationships play a crucial role in determining how talent is perceived and acknowledged. Cattani et al. (2014) extend this study by drawing on field theory to hypothesize that peers in the film industry favor creators at the core of the collaboration network, while film critics may prefer those at the periphery. These hypotheses are tested using a dataset of over 12,000 crew and cast members who worked on movies distributed by eight major studios in the United States from 1992 to 2004. The dataset includes information on directors, writers, leading and supporting actors/actresses, editors, cinematographers, and production designers, sourced from IMDb. They use a discrete choice model to analyze award decisions by organizations and estimate the choice set of eligible professionals. Collaboration networks are created based on person-film affiliations and analyzed using a core-periphery algorithm. Node classification as core or periphery is then used as a predictor in the models. Results show that being in the periphery negatively affects peer recognition but has a slight positive association with recognition by critics, controlling for other factors.

The impact of network closure on gender inequality in a project-based labor market was examined in an important study by Lutter (2015) of Hollywood film productions between 1929 and 2010. Lutter analyzes data on 1,072,067 film performances by 97,657 actors, $30\%$ of which are women, taken from IMDb. The author utilizes Cox regression models, a type of survival analysis, to estimate the risk of career failure, represented as a binary variable in which a value of 1 indicates an actor’s final appearance. The results find that gender equality is more attainable in a network structure that is open and diverse, leading to statistically insignificant differences between men and women in terms of career failure when there is no team cohesion present. Verhoeven et al. (2020) build on Lutter’s research on gender inequality in the film industry and analyze the role of social networks in perpetuating this inequality. The study uses data from the Australian, German and Swedish film industries and confirms previous findings that women tend to remain on the periphery of networks unless they can create ties with key players. The authors further propose control mechanisms to address this, such as establishing employment connections between women and men who are key players, but acknowledge that these interventions may not always be desirable in practice. The authors argue that aggregated statistics on gender inequality are not enough to propose meaningful change and that research should focus on understanding gender inequality in terms of uneven social relations.

Advancing the analysis of gendered social relations in the film industry, Fanchi and Tarantino (2020) provide an analysis of the impact of digital transformation on the Italian cinema industry, particularly on the position of women in production structures. They find that women have a peripheral role in the Italian cinema industry and are less likely to work on films with higher budgets and prestige. They note that women are numerically underrepresented, but that the problem of women creatives in the industry is not just about number but also power and position; women are not only excluded, those that are able to enter the industry occupy less central positions. This imbalance can have cultural, symbolic, and economic repercussions on industry competitiveness and renewal. Overall, the study suggests that digital transformation has not been sufficient to eradicate the gendered logics of Italian cinema, and bolder interventions are necessary to promote a more inclusive production system. The paper proposes that policy reforms need to place more emphasis on connecting the clusters of women creatives with the larger and more powerful regions of the Italian film industry creative network.

Juhász et al. (2020) combine gatekeeping analysis with a focus on creative success to examine how core creators benefit from brokerage in core/periphery networks using a unique, open access dataset of Hungarian feature films. They find that creators who bridge the core and periphery in the collaboration network have the highest likelihood of creative success. This dual core-broker position provides complementary benefits to central creators. The study challenges the core/periphery trade-off using a measure called gatekeeping to identify brokers between the core and the periphery of the network. The analysis is based on the three-way interaction effects of coreness, edge betweenness-based brokerage, and gatekeeping to understand their joint impact on creative success.

#### Specific industry studies

Two papers analyse the adult film industry as a specific case study. Gallos et al. (2013) utilize the movie co-appearance network from IMDB, in which actors are connected if they have appeared in the same film, to demonstrate how to uncover fractality in a small-world network. They analyzed a subset of the IMDB network containing “Adult” films, arguing that this subset demonstrates a clear transition from small-world to fractal and modular structures because the genre is more recent, more focused in time, and isolated from other genres. The study uses the box-covering technique and renormalization to conduct the analysis. Results show that the strength of links, or the number of times actors collaborate, can significantly impact the network topology. When all actors are linked, the network becomes small-world and lacks a proper modular structure. However, when a threshold is imposed on the minimum number of links between two actors, the network becomes naturally fractal. Lüdering (2018) also examines adult film industry networks to evaluate the link between collaboration network position and career duration in the adult film industry. Using data from the Internet Adult Film Database, Lüdering creates yearly networks of adult film performers based on co-appearance in films and then, drawing from social capital theory, applies eigenvector centrality to analyze how network positioning impacts career length. Survival models include centrality, connection to the main network component, age at entry, and gender as explanatory variables. Different approaches to baseline hazard distribution are considered, including stratified/unstratified Cox regressions and parametric models. In all cases, centrality lowers exit risk, while disconnection from the main network raises it.

There are a series of studies that delimit their exploration to the networks found within national cinemas. Two interrelated studies use social network analysis to study the role of key individuals in the Afrikaans film industry. Senekal and Stemmet (2014) analyse the structural importance of filmmaker Jamie Uys in the industry, while Senekal (2014) focuses on director Pierre de Wet. Both papers use the same data set on the Afrikaans film industry and deploy very similar analytical methods. The dataset for the Afrikaans film industry includes entities such as films, directors, film editors, producers, writers, cinematographers, music composers, production companies, and distribution companies from 1916 to 2013. The initial information was obtained from a 1982 study by Le Roux and Fourie, and additional details were then gathered from IMDb and production company web archives. The different entity types form a multimodal network, and all analyses are based on different subsets of this network. Senekal finds that Pierre de Wet had a high level of centrality and was able to spread knowledge throughout the industry network. Senekal argues that the high degree centrality of de Wet and many of his collaborators had a significant impact on the flow of information in the film industry as it allowed for the accumulation of experience through working on a large number of films, as well as the transfer of experience through contact with many other individuals who also have many connections. Similarly, Senekal and Stemmet find that both Uys and his films have high betweenness centrality, making him a hub in the industry.

Two papers in our survey focus specifically on the Turkish film industry. Abanoz (2020) uses data from IMDb to explore the small world properties of the actor co-occurrence network in Turkish cinema. The study uses closeness and average path length to identify the actors who are at the centre of the small world structure in the one-mode actor-actor projection of the actor-film affiliation network. They find that Süheyl Eğriboz is the most central by these measures and argue that he was the Turkish equivalent of Kevin Bacon in that everyone in Turkish cinema is either directly connected with him or indirectly connected to him through few intermediary collaborators. In another paper examining Turkish film industry networks, Savk and Dogu (2021) critique the traditional approach to film historiography that focuses on films and major players. Using the Yeşilçam subindustry in the 1960s and 1970s as a case study, the authors employ network analysis to shift the focus onto the connections among writers, directors and producers based on patterns of collaborating on films. They identify two distinct clusters in the resulting network visualizations, which they argue accurately reflect the split between mainstream and lower-budget modes of production in that era.

Neuberger (2020) explores a network depiction of the Soviet film industry from 1918 to 1953. Using data from the Russian film database kino-teatr.ru, the author scrutinizes director-actor connections primarily using eigenvector centrality and community detection. The research reveals that the director-actor network starts with divisions along ethnic and geographic lines but gradually integrates over time. Furthermore, the author notes that those with high eigenvector centrality are not the typical figures identified as significant by Soviet cinema scholars and argues that a network approach provides a distinct viewpoint on the composition and development of the Soviet film industry.

Finally, Noroozian et al. (2022) analyze an original dataset of Iranian actors who participated in the Fajr Film Festival from 1998 to 2020 to investigate yearly collaboration networks among the actors. Their analytical focus is on the decreasing diversity of Iranian film genres over time and the impact of persistent collaboration between key actors on audience preferences, stability of genres, and box office measures. Findings suggest that network density negatively affects box office, but one particular type of film, the social problem genre, has stabilized due to the continuous cooperation between core actors. Furthermore, there is a positive correlation between the importance of actors in the network and their popularity on Instagram, indicating that people follow those central in the actors’ network.

Table 1 highlights some of the main features of the research efforts reported in this section. We list the reported size of the network(s) analyzed in each reviewed paper as well as the availability of the data in order to aid researchers in identifying the network data that may be most useful for further analysis.

**Table 1:** Feature summary of reported film network analysis with people as nodes

References	Data size^a^	Evaluation methodology
Faulkner and Anderson (1987)	2, 430 films	Regression
Jones (1996)	606 films	Component, *k*-core analysis
Jones and Walsh (1997)	606 films	Component, *k*-core analysis
Delmestri et al. (2005)	705 films	Regression
Ferriani et al. (2005)	762 feature films	Blau’s index, t-test
Perretti and Negro (2007)	6, 446 feature films	Regression
Cattani and Ferriani (2008)	2, 137 films	Regression
Krauss et al. (2008)	-	Correlation analysis
Rossman et al. (2010)	16, 392 films	Regression
Pontikes et al. (2010)	5, 712 feature films	Weighted logit regression
Ebbers and Wijnberg (2010)	233 films	Correlation analysis
Meiseberg and Ehrmann (2013)	180 films	Regression
Gallos et al. (2013)	39, 397 adult films	Renormalization group analysis
Cattani and Ferriani (2013)	2, 297 films	Logit regression
Cattani et al. (2014)	2, 297 films	Logit regression
Senekal and Stemmet (2014)	–	Centrality analysis
Senekal (2014)	–	Centrality analysis
Viana et al. (2014)	3, 006 films	Network analysis
Lutter (2015)	369, 099 films	Regression
Negro and Goodman (2015)	–	Multi-level logit regression
Lüdering (2018)	102, 871 adult films	Survival analysis, regression
Juhász et al. (2020)	7, 672 creators	Regression, centrality analysis
Fanchi and Tarantino (2020)	3, 542 films	Centrality analysis
Verhoeven et al. (2020)	4, 054 creators	Network analysis
Abanoz (2020)	9, 441 films	Network analysis
Neuberger (2020)	4, 810 directors/actors	Network analysis
Savk and Dogu (2021)	5, 595 films	Network analysis
Karpov and Marakulin (2022)	85, 855 films (Y)	Network and predictive analysis
Noroozian et al. (2022)	–	Network analysis

$^{a}$ Refers to the target network and whether the dataset used is publicly available (Y)

### Node type: films

#### Critical success indicators

Less prevalent than people nodes but still significant, is the use of film titles as network nodes. Much of this research focuses on measuring the success and/or impact of particular films or collections (e.g. genres) of films. Meiseberg and Ehrmann (2008) provide an early example of this approach using data on the German film industry. Their dataset is drawn from the 10 highest-admission German films each year as recorded by the German national film funding agency, amounting to 111 films released between 1993 and 2004. From these data they first construct two-mode actor-movie networks, which they project down to a one-mode movie-movie network in which ties represent shared actors. They conduct regression analyses to test hypotheses about the effects of various film-level factors on the performance of German films, measured in terms of admissions. They include variables in this model for the degree of movies and the local clustering coefficient in the movie-movie network. They find that degree - i.e. the number of movies with which the focal movie shares actors - is positively associated with movie success, while there is no significant effect for the local clustering coefficient.

Seeking to understand the context for different kinds of film success, Miller (2011) compares the funding models and network patterns of commercially successful and critically acclaimed US films. The study scrutinizes co-production network structures and how they evolve with production changes focusing on high-quality film funding and network structures in domestic and international settings. The role of inter- and intra-nationality in company relationships and their impact on film success is explored. The paper’s findings show that the network structures of both commercially successful films and critically acclaimed ones are significantly similar, being less centralized, clustered and dense than anticipated. However, critically acclaimed films exhibit greater collaboration and network “penetrability” compared to their high-grossing counterparts.

#### Interconnected movies

A notable group of film-oriented network papers explore the impact, influence and interconnection of films with one another through “on-screen” indicators of intertextuality. These papers primarily draw on the user-submitted “Connections” field on a film’s IMDb page, which allows users to establish six types of connections among movies, including features, references, follows, spoofs, remakes, and spin-offs. For example, Spitz and Horvát (2014) use this information to construct a network of film “citations” to evaluate cinematic impacts and milestones. Films are represented as nodes and citations as edges. Centrality indices, such as weighted out-degree, temporal degree, influence time, subtree, start, and propagation, are used to measure impacts. The films are then ranked based on their position in the network and compared to various “greatest film” lists. The authors find that films with long-term impact, as measured by film citations, do not always correspond to high ratings or awards.

Wasserman et al. (2014) explore the correlation between IMDb user scores and film budgets and gross earnings. The authors construct a film network based on IMDb’s “Connections” section for each film. During network construction, they notice a significant bias toward U.S. films in terms of incoming and outgoing connections. To mitigate this bias, they narrow their dataset to exclusively include U.S. films thereby avoiding any confounding factors associated with the country of production. Their findings reveal a strong correlation between the number of votes and economic statistics, particularly the film’s budget. They conclude that the quantity of ratings is a reliable indicator of a film’s prominence.

Also seeking to explain film success, Bioglio and Pensa (2017) introduce the “influence score” as an innovative metric for evaluating a movie’s performance. This method differs from traditional approaches reliant on economic sales data or subjective critic assessments, which can be influenced by marketing strategies, inflation, diverse distribution platforms, and personal biases. The influence score hinges on a film’s ability to inspire subsequent works through creativity, innovation, and franchise-building. The study investigates intricate patterns and trends related to a film’s release year, genre, and production country. Again, the authors define a citation network where nodes represent movies, connected by directed edges whenever a connection (per IMDb) exists. The study employs centrality scores, including in-degree, closeness, harmonic, and PageRank measures, implemented using Python and the NetworkX library, to evaluate the network’s dynamics. The findings indicate that the top 20 influential movies, based on influence centrality, predominantly originate from the pre-1940 era, highlighting the enduring impact of classic films. The authors identify that movies from the United States are the most influential overall, while French, Italian and German films have also proved influential in different historical periods. In a related publication, Bioglio and Pensa (2018) argue that citations between films offer a more dependable measure of a film’s impact than assessments by film critics, economic data, or historical comparisons.

Shin et al. (2022) develop a variation of “citation” measures by examining the evolution of movie genres and subgenres and their influence on popular culture through the analysis of memes as indicators. They introduce the “meme score”, determined by machine learning algorithms analyzing user reviews and ratings for both movies and tags. Each tag is treated as a meme, comprising elements of a film. Networks of movies are then created by connecting films with similarities that exceed a certain threshold. The study reveals that cross-genre connections grow over time, with successful films spawning new memes. However, the methodology lacks clarity regarding the precise generation of these networks, aside from the use of ML algorithms.

Film industries are notoriously risk averse and the ability to predict box office is a longstanding research problem. Yahav (2016) emphasizes the importance of understanding consumer choice in predicting movie demand, asserting that movies with similar appeal should exhibit similar demand patterns. To enhance box office success forecasting, they introduce an automated technique that leverages a similarity network. This technique measures several aspects of demand structures, including decay rate, time of the first demand peak, per-screen gross value at peak time, existence of a second demand wave, and time on screens. Their argument highlights the superior predictive power and robustness of models incorporating a similarity network compared to those that do not. Table 2 summarizes key features of the research endeavors discussed in this section.

**Table 2:** Feature summary of reported film network analysis with films as nodes

References	Data size	Evaluation methodology
Meiseberg and Ehrmann (2008)	111 films	Network analysis, OLS regression
Miller (2011)	110 films	Network and correlation analysis
Wasserman et al. (2014)	32, 636 films	Correlation analysis
Spitz and Horvát (2014)	40, 000 films	Centrality analysis
Yahav (2016)	2, 240 films	Regression
Bioglio and Pensa (2017)	65, 000 films	Centrality analysis
Bioglio and Pensa (2018)	47, 000 films	Centrality analysis
Shin et al. (2022)	10, 380 films	Machine learning

### Node type: companies, geographical units, and others

In this section, we compile research papers whose network nodes represent companies, geographical units, or other relevant entities in the film industry. These papers are summarized in Table 3.

#### Networks and box-office prediction

The interest in the value of networks for predicting film industry performance continues in this section with Doshi et al. (2010) who explore the effectiveness of social network analysis and sentiment analysis within the context of predicting Hollywood Stock Exchange (HSX) pricing trends during the first four weeks following a movie release. This prediction process operates by predicting the potential financial success of the film, predicting the audience’s reception and likability of the film, and estimating the film’s actual earnings within the first month after its release. To facilitate these predictions, the authors harness web metrics extracted from IMDb, Rotten Tomatoes, movie quotes from HSX, and box office collection data from Box Office Mojo. They conduct sentiment analysis on forum posts within IMDb to discern positive and negative connotations and assess the film’s popularity. Additionally, they gather data on movie titles and monitor their relevance within the blog space, termed the “information sphere”. Utilizing this data, they calculate betweenness centrality to quantify the buzz and attention a movie garners on the Internet. To refine their predictions, a combination of multilinear and non-linear regression models is employed. The paper ultimately establishes a predictive model capable of predicting the flops and blockbusters with few discrepancies.

#### Film production clusters

Most papers in this section however are concerned with the organization of the film production sector. In an early network analysis, Krätke (2002) delves into the examination of production clusters within the Potsdam/Babelsberg region of Germany. The primary emphasis lies in utilizing networks as a methodological approach for scrutinizing regional clusters. Through a comprehensive survey, the author identified a total of 46 companies which comprise the network nodes. The study assesses seven cluster attributes, encompassing functional differentiation, network density, network cohesion, network centralization, supra-regional transactions, the spatial density of cluster firms, and the quality of the cluster’s institutional infrastructure. The paper concludes that the Potsdam/Babelsberg region aligns with the concept of an “ideal cluster”, and suggests that the network-based methodology provides useful analytical criteria for the evaluation of production clusters. Xin and Mossig (2020) also look at networks of production companies in the top-grossing films from Germany (2012–2017), comparing them to company networks in China (2002–2017). Their interest is in the role of formal institutions, including state actors, in the film production process, and they compare the contrasting economic contexts of Germany and China. As well as the production company network, they look at the location of the companies and create an aggregated network of geographic nodes. They find that formal institutions play a stronger role in the Chinese production network than the German one, in line with their expectations.

Zhou (2022) also investigates the structure of the Chinese film industry from a network perspective. Using data on the top 20 grossing Chinese films from 2010-2020, the author performs some simple graph analysis of three related networks: the two-mode film director to production company network, the one-mode company-level projection, and the one mode city-city network based on the locations of companies in the company network. They calculate centrality measures and identify cohesive subgroups to understand the structure of these networks, concluding that the networks are characterized by power imbalances, as a “minority of directors and companies dominate the majority of film resources”. They find that these dominant players primarily represent production cultures in the traditionally dominant economic centers of Hong Kong, Beijing and Shanghai. However, based on the spatial patterns in the city network, they suggest that China’s shifting economic landscape has possibly begun to see influence dispersed throughout several emerging eastern cities, dislodging the dyadic control of Beijing and Shanghai.

Cattani et al. (2008) investigate how network structures of organizations in the film industry might be linked to consensus and legitimacy among production companies. The authors put forth the following hypotheses: (i) greater network connectivity will diminish the exit rates of production companies. This arises from the idea that well-connected networks tend to converge on shared understandings, thanks to reduced gaps in knowledge and information diversity; (ii) repetitive interactions between organizations will likewise reduce the exit rates of production companies. This results from the formation of norms through trial and error, where repeated interactions increase opportunities for these normative processes; and (iii) a higher turnover rate among distribution companies will increase the exit rates of production companies. This stems from the potential for newcomers to disrupt stability and shared understandings. The study tests these hypotheses by constructing a network that represents production companies and the distributors with which they collaborated on films, covering the period from 1912 to 1970 in Hollywood. The resulting network statistics are then used as independent variables within a survival model for the production companies, ultimately substantiating the proposed hypotheses.

#### Co-productions as networks

Two papers further examine co-production networks. Hoyler and Watson (2018) investigate urban relationships by applying network analysis to co-productions within the film industry. They focus on four key markets: China, Germany, Brazil, and France. The study involves collecting data on production companies and their locations from the top 200 highest-grossing films in 2013 and 2014. Using Gephi, they employ community detection and degree centrality to measure node importance. The findings indicate that film production networks are shaped by pre-existing city relationships. France exhibits a monocentric structure, while Brazil and China have a dyadic pattern, and Germany is characterized by a polycentric model. Additionally, established film production hubs such as Los Angeles, London, and Tokyo are consistently present in all analyzed networks. Zhang and Wang (2020) examine the influence of the Chinese “Closer Economic Partnership Arrangement” (CEPA) on co-production networks between Mainland China and Hong Kong. Their simple network analysis revealed that a few studios in both regions held dominance in terms of the number of films produced, degree centrality, and betweenness centrality. They argue that the less centralized filmmaker collaboration network portrays a scenario in which Mainland China, primarily through producers, wields more control over the financial and administrative aspects of the industry, while Hong Kong, particularly through directors, exerts greater creative and artistic influence.

#### Distribution networks

da Rocha et al. (2018) analyze the Brazilian film industry using social network analysis to evaluate the relationships between production and distribution companies. To construct the network, the authors identified film production and distribution companies with associated films released between 1995 and 2015. They calculate measures of cohesion (density, centralization) and centrality (degree, betweenness, closeness). Despite assessing 613 production companies, only a handful of these companies have cooperative relationships with each other. The lack of cooperative relationships between companies is generally present throughout the results, suggesting that the overall industry in Brazil is fairly restricted and dispersed. In their 2019 study, Zhang et al. (2019) also analyze project alliance networks in the film industry using a dataset of production and distribution companies. They sought to understand how alliance network characteristics influenced project performance, specifically examining company-level alliances in both production and distribution. Employing a regression model with box office performance as the dependent variable and network properties as independent variables, the study made several key findings: financial success correlated significantly with alliance network properties, increased degrees of production companies in production alliances positively impacted box office performance, while the degrees of distribution companies in distribution alliances had no significant influence. Interestingly, eigenvector centrality and betweenness centralities in both types of networks did not strongly affect box office performance. Notably, structural holes in distribution alliance networks had a positive impact on box office success, while those in production alliance networks did not. In conclusion, this research highlights the differing effects of network properties in production and distribution alliances on box office performance, with production alliances exerting a stronger influence.

Choi et al. (2012) are also interested in investigating the intricate system of international film distribution, treating it as a trade network with the objective of shedding light on the flow of film products among developed nations. They discern whether there is a discrepancy between imports and exports in international trade, whether there is a temporal pattern to the networks, and whether there is a geolinguistic clustering among countries in the networks. To test these research questions, they use OECD data on international trade to construct adjacency matrices representing imports and exports of films between countries in two time periods: 1996-2001 and 2002-2006. These networks contain 32 nodes including 30 OECD countries plus China and Hong Kong. They use QAP correlation, eigenvector centrality, multidimensional scaling (MDS) and cluster analysis to analyze their trade networks. They find a moderate to large asymmetry in the import and export networks which is larger in the second time period than the first. In both exports and imports, the US and Canada had the highest centrality scores, and the centrality score ranks were highly correlated between the two time periods, especially for exports. Their MDS reveals close links in all cases between the US and Canada, and among the United Kingdom and France in the import networks, with Asian and Eastern European countries as well as Mexico appearing on the periphery. Their hierarchical clustering analysis (which deliberately omitted the United States) found a group of Commonwealth countries, an East Asian group and smaller European clusters in the early export network, while the later export network was split between a European group (with Canada) and an East Asian group. In a similar vein, Coate et al. (2016) use a large global dataset of cinema showtimes to analyze the reciprocity of cultural exchanges by calculating the import and export of films at the national level. The study is particularly interested in reciprocity as a largely overlooked element in understanding cultural exchange and diversity. Informed by digital humanities, network analysis, cultural economics and geo-spatial sciences, the authors highlight those national relationships of exchange that cross-cut traditionally perceived unilateral cultural flows. They find that countries that are close to each other in terms of language, population size and economic development are more likely to have a more equitable and reciprocal relationship in the cinematic exchange network.

Also focusing on distribution networks, Ehrich et al. (2022) depict the film festival sector as a network, with festivals connected via film flow (one-mode network) or films linked to festivals (two-mode network). This analysis targets the structural persistence of gender inequality in the sector, using data from 1,523 film festivals and 1,323 films in the 2013 circuit, collected from the “Film Circulation in the International Festival Network and the Influence on Global Film Culture” project. Gender is attributed to a film based on the core creative (writer, director, producer) team's gender ratio, determined computationally using GenderizeR and Gender Guesser. The study views festivals as network agents (nodes) and films as connections, focusing on aggregate social structures rather than individuals. By examining degree distributions among films from women-only, men-only, and mixed-gender core creative teams across festivals and comparing various statistical measures, the study reveals that festivals screening women-led films exhibit less overlap in their film selections compared to those showcasing predominantly male-led films. A select few films from male-led teams circulate more successfully across festivals.

#### Reward networks

In a unique study that proposes film awards as a node, Liu and Ma (2022) examine the dynamics of the film industry’s award system to not only explore the growth of the award system itself, but to also understand what leads to a performer’s early career success and initial nominations. They create two networks: an award network (where awards are nodes and the links the number of performers winning both awards), and a performers’ collaboration network. They utilize a logistic regression model to predict a performer’s propensity of prize-winning (the probability that they will win at their first nomination). Their findings show, firstly, that historical performance positively influences an actor’s future award-winning. Furthermore, the longer the performer’s career, the higher likelihood that they will win awards. The second main finding is the performer’s collaborator influence. Actors who had already won awards had a positive effect on a performers’ prize-winning. However, collaborating with directors had a negative effect. The authors conclude that their results indicate that “success breeds success” in the film industry.

**Table 3:** Feature summary of reported film network analysis with other entities as nodes

References	Data size	Evaluation methodology
Krätke (2002)	55 companies	Network analysis
Cattani et al. (2008)	–	Regression
Doshi et al. (2010)	–	Regression
Choi et al. (2012)	32 countries	Network analysis, correlation analysis
Coate et al. (2016)	–	Network analysis
da Rocha et al. (2018)	613 companies	Centrality analysis
Hoyler and Watson (2018)	–	Network analysis
Zhang et al. (2019)	86, 503 companies	Network analysis, regression
Zhang and Wang (2020)	243 studios	Network analysis
Xin and Mossig (2020)	460 companies	Network analysis
Ehrich et al. (2022)	1, 523 film festivals	Network analysis
Liu and Ma (2022)	3, 634 awards	Network analysis, regression
Zhou (2022)	47 cities, 455 companies	Network analysis

## Discussion and conclusions

There are several preliminary conclusions we can draw about the application of network science and theory to film industry analysis and in this context, we also propose avenues for future research. First, our survey reveals that in the initial 25 years of film network scholarship, analysis has focused primarily on networks of individuals and, to a lesser extent, films. In more recent years, a notable diversification has since transpired, and the analytical scope has expanded to include production companies, geographic units, and various hybrid combinations of these entities. As such, film network research is now able to offer a broader and more comprehensive understanding of the complex network dynamics within the film industry. This is promising, but more integration of theory from the relevant domains is needed to push the network analysis beyond simple descriptions of static network structure.

Second, we note that a disproportionate amount of the literature (especially the formative literature for theory-building in this area) is focused on the film industry of the United States, and more work is needed to see whether other film industries are governed by the same network principles. This focus on Hollywood may well be related to the geographic profile of who is contributing to this emerging literature, as a review of the institutional affiliations of authors shows that this literature is dominated by scholars from the Minority World (particularly the United States, Europe and Australia). In this respect, it is encouraging to see that in recent years there has been a growth in the number of contributions to this research area from authors in the Majority World, including analyses of film industries outside of the dominant global export markets. However, we note that the flow of recognition and scholarly engagement is uneven, with authors in the Minority World citing the contributions of scholars in the Majority World far less than they are cited by them.

Third, many of the studies in this survey use historical data and their conclusions can be re-evaluated with more recent data. The film industry and its practices shift over time and should be re-evaluated empirically. Studies can also be examined to test the reproducibility of results. Related to this point, only a few of these studies have made their datasets available. More research in this space could embrace open science principles, especially given that the datasets are typically constructed from public sources with minimal ethical concerns.

Fourth, our review highlights that, with a few valuable exceptions, very little scholarship has been done which looks at inequalities in the film industry from a relational perspective. Qualitative and non-relational quantitative evidence (not reviewed in this paper) persistently highlights how inequitable these industries and their workforces are. Moreover, the foundational work we reviewed by Faulkner and Jones provides rich evidence of how important reputational and relational mechanisms are for structuring opportunities in project-based cultural industries. These factors intersect with known network mechanisms of exclusion such as homophily and familiarity to systemically disadvantage some people and benefit others. Despite this, relatively few papers take a network analytic approach to studying film industry inequalities, which in turn makes it harder to develop evidence-based policies that aim to redress the industry’s uneven relationships.

Fifth, much of the literature relies on one-mode projections of co-affiliation data. Primarily, scholars have analyzed the people-to-people projection of the co-affiliation data, though some papers have analyzed the film-to-film projection as well. Each of these projections offers a limited understanding of the personal networks that organize the industry, or the intertextual structures that emerge from the flow of people between film projects. More methodological sophistication in addressing the inherent multimodality of this type of data is needed. More work is also needed which accounts for the dependencies between different types of nodes (e.g. people and places) that comprise film industry network structure. While some recent contributions have analyzed different types of nodes within the same study, these analyses have largely treated each type of node separately rather than aiming to represent them within a complex multi-level network structure. We hope that scholars will use advances in multi-level network methodology and theory to help model the multi-level nature of actors within the global film industries.

Finally, more engagement with existing scholarship is needed, especially among researchers from computational disciplines who wish to use the film industry as a case study for advanced methods. There is enough literature that we should expect more cross-citation among the contributions. However, papers continue to appear which apply network analysis to the film industry and yet do not acknowledge any of the earlier work in this area. For the research area to continue to develop, scholars need to properly recognize, engage with and build on the vast amount of scholarship already undertaken. We hope that this survey paper can help simplify this process for scholars wishing to contribute further to this fast-growing topic.

Together, these findings contribute a synthesis of an important and popular area of network science application that has not yet been systematically reviewed to find coherent themes and gaps. We have highlighted several ways in which applied network scientists can advance understanding of the film industry, and flagged areas that our survey reveals as being in particular need of more attention. There are also several limitations to the present survey. Firstly, the restrictions we placed on our literature search, including the decision to only review publications in English, helped ensure that a survey was feasible and bounded, but it inevitably means that we will not have included some valuable contributions in this space. Furthermore, as the global film industry grapples with technological, business and social challenges we acknowledge the types of nodes we have outlined and their relational dimensions will also evolve and we look forward to the terms of this survey being extended in future iterations of film industry network analysis.

## Data Availability

Not Applicable

## References

[CR1] Abanoz E (2020) The degrees of separation in Turkish cinema: The Sühely Eğriboz number. J Erci Commun 7(2):1199–1212. 10.17680/erciyesiletisim.708514

[CR2] Ahmed A, Batagelj V, Fu X, Hong SH, Merrick D, Mrvar A (2007) Visualisation and analysis of the Internet movie database. In: Proceedings of the 6th International Asia-Pacific Symposium on Visualization, pp 17–24

[CR3] Amaral LAN, Scala A, Barthélémy M, Stanley HE (2000) Classes of small-world networks. Proc Natl Acad Sci 97(21):11149–11152. 10.1073/pnas.200327197 11005838 10.1073/pnas.200327197 PMC17168

[CR4] Barabási AL, Albert R (1999) Emergence of scaling in random networks. Science 286(5439):509–512. 10.1126/science.286.5439.509 10521342 10.1126/science.286.5439.509

[CR5] Bioglio L, Pensa RG (2017) Is this movie a milestone? Identification of the most influential movies in the history of cinema. Complex Networks & Their Applications. Springer, London, pp 921–934. 10.1007/978-3-319-72150-7_74

[CR6] Bioglio L, Pensa RG (2018) Identification of key films and personalities in the history of cinema from a Western perspective. Appl Netw Sci 3(1):1050. 10.1007/s41109-018-0105-0 10.1007/s41109-018-0105-0 PMC6276061 30596143

[CR7] Cattani G, Ferriani S (2008) A core/periphery perspective on individual creative performance: Social networks and cinematic achievements in the Hollywood film industry. Organiz Sci 19(6):824–844. 10.1287/orsc.1070.0350

[CR8] Cattani G, Ferriani S (2013) Networks and rewards among Hollywood artists: Evidence for a social structural ordering of creativity. In: Kaufman J, Simonton D (eds) The Social Science of Cinema. Oxford University Press, pp 185–206. 10.1093/acprof:oso/9780199797813.001.0001

[CR9] Cattani G, Ferriani S, Allison PD (2014) Insiders, outsiders, and the struggle for consecration in cultural fields. Am Sociolog Rev 79(2):258–281. 10.1177/0003122414520960

[CR10] Cattani G, Ferriani S, Negro G, Perretti F (2008) The structure of consensus: Network ties, legitimation, and exit rates of U.S. feature film producer organizations. Administr Sci Quart 53(1):145–182. 10.2189/asqu.53.1.145

[CR11] Choi JH, Lee SW, Chon BS (2012) Transitions in the film trade among OECD countries: A network approach. Media Int Australia 142(1):16–29. 10.1177/1329878x1214200104

[CR12] Coate B, Verhoeven D, Arrowsmith C, Palmer S (2016) Using big cultural data to understand diversity and reciprocity in the global flow of contemporary cinema. pp. 141–151. UNESCO Institute for Statistics

[CR13] Coe NM (2000) The view from out west: embeddedness, inter-personal relations and the development of an indigenous film industry in Vancouver. Geoforum 31(4):391–407. 10.1016/s0016-7185(00)00005-1

[CR14] da Rocha DT, Bonfim LRC, Citadin MW, Gimenez FAP (2018) Mapping co-production and co-distribution relations in the Brazilian cinema: an analysis under the light of the network theory. Intercom Revista Brasileira de Ciências da Comunicação 41(1):41–61. 10.1590/1809-5844201812

[CR15] Delmestri G, Montanari F, Usai A (2005) Reputation and strength of ties in predicting commercial success and artistic merit of independents in the Italian feature film industry. J Manag Stud 42(5):975–1002. 10.1111/j.1467-6486.2005.00529.x

[CR16] DiMaggio P (2011) Chapter 20: cultural networks. In: Scott J, Carrington P (eds) The Sage handbook of social network analysis. SAGE Publications Ltd., United Kingdom, pp 286–301

[CR17] Doshi L, Krauss J, Nann S, Gloor P (2010) Predicting movie prices through dynamic social network analysis. Proced Soc Behav Sci 2(4):6423–6433. 10.1016/j.sbspro.2010.04.052

[CR18] Ebbers JJ, Wijnberg NM (2010) Disentangling the effects of reputation and network position on the evolution of alliance networks. Strat Organiz 8(3):255–275. 10.1177/1476127010381102

[CR19] Ehrich ME, Burgdorf K, Samoilova Z, Loist S (2022) The film festival sector and its networked structures of gender inequality. Appl Netw Sci 7(1):457. 10.1007/s41109-022-00457-z

[CR20] Fanchi M, Tarantino M (2020) Has digital transformation impacted gender imbalance in Italian cinema? A data analysis of creative clusters 2004–2016. In: Massi M, Vecco M, Lin Y (eds) Digital transformation in the cultural and creative industries: production, consumption and entrepreneurship in the digital and sharing economy. Routledge, Oxford-New York, pp 162–84

[CR21] Fass C, Turtle B, Ginelli M (1996) Six degrees of Kevin Bacon. Plume, New York

[CR22] Fatemi M, Tokarchuk L (2012) An empirical study on IMDb and its communities based on the network of co-reviewers. In: Proceedings of the 1st Workshop on Measurement, Privacy, and Mobility, pp 1–6

[CR23] Faulkner RR (1983) Music on demand: Composers and careers in the Hollywood film industry. Transaction Books, New Brunswick, NJ

[CR24] Faulkner RR, Anderson AB (1987) Short-term projects and emergent careers: Evidence from Hollywood. Am J Soci 92(4):879–909. 10.1086/228586

[CR25] Ferriani S, Corrado R, Boschetti C (2005) Organizational learning under organizational impermanence: Collaborative ties in film project firms. J Manag Govern 9(3–4):257–285. 10.1007/s10997-005-7422-2

[CR26] Gallos LK, Potiguar FQ, Andrade JS, Makse HA (2013) IMDB network revisited: Unveiling fractal and modular properties from a typical small-world network. PLoS ONE 8(6):e66443. 10.1371/journal.pone.0066443 23826098 10.1371/journal.pone.0066443 PMC3691214

[CR27] Hoyler M, Watson A (2018) Framing city networks through temporary projects: (Trans)national film production beyond ‘Global Hollywood’. Urban Stud 56(5):943–959. 10.1177/0042098018790735

[CR28] Jones C (1996) Careers in project networks: The case of the film industry. In: Arthur MB, Rousseau DM (eds) The Boundaryless career: A new employment principle for a new organizational era. Oxford University Press, Oxford, pp 58–75

[CR29] Jones C, Walsh K (1997) Boundaryless careers in the us film industry: Understanding labor market dynamics of network organizations. Ind Bezieh German J Ind Relat 4(1):58–73

[CR30] Juhász S, Tóth G, Lengyel B (2020) Brokering the core and the periphery: Creative success and collaboration networks in the film industry. PLOS ONE 15(2):e0229436. 10.1371/journal.pone.0229436 32106266 10.1371/journal.pone.0229436 PMC7046270

[CR31] Karpov I, Marakulin R (2022) Social network analysis of the professional community interaction—movie industry case, Communications in Computer and Information Science, 36–50. Springer International Publishing

[CR32] Krauss J, Nann S, Simon D, Gloor PA, Fischbach K (2008) Predicting movie success and academy awards through sentiment and social network analysis. In: Proceedings of the 16th European conference on information systems

[CR33] Krätke S (2002) Network analysis of production clusters: The Potsdam/Babelsberg film industry as an example. Europ Plann Stud 10(1):27–54. 10.1080/09654310120099254

[CR34] Labatut V, Bost X (2019) Extraction and analysis of fictional character networks. ACM Comput Surv 52(5):1–40. 10.1145/3344548

[CR35] Liu Y, Ma Y (2022) Quantifying award network and career development in the movie industry. Front Phys 10:890. 10.3389/fphy.2022.902890

[CR36] Lutter M (2015) Do women suffer from network closure? The moderating effect of social capital on gender inequality in a project-based labor market, 1929 to 2010. Am Sociolog Rev 80(2):329–358. 10.1177/0003122414568788

[CR37] Lüdering J (2018) Standing and ‘survival’ in the adult film industry. Appl Econom 50(16):1812–1823. 10.1080/00036846.2017.1374542

[CR38] Madduri K, Ediger D, Jiang K, Bader DA, Chavarria-Miranda D (2009) A faster parallel algorithm and efficient multithreaded implementations for evaluating betweenness centrality on massive datasets. In: Proceedings of the IEEE international symposium on parallel & distributed processing, pp 1–8

[CR39] Meiseberg B, Ehrmann T (2008) Performance implications of network structure, resource investment, and competition in the German motion picture industry, Contributions to Management Science. 347–372. Physica-Verlag HD

[CR40] Meiseberg B, Ehrmann T (2013) Diversity in teams and the success of cultural products. J Cultl Econom 37(1):61–86. 10.1007/s10824-012-9173-7

[CR41] Miller JL (2011) Producing quality: A social network analysis of coproduction relationships in high grossing versus highly lauded films in the US market. Int J Commun 5:20

[CR42] Negro G, Goodman S (2015) Niche overlap and discrediting acts: An empirical analysis of informing in Hollywood. Sociolog Sci 2:308–328

[CR43] Neuberger J (2020) Centrality and centralisation a social network analysis of the early Soviet film industry, 1918-1953. Apparatus. Film, Media and Digital Cultures of Central and Eastern Europe 10. 10.17892/APP.2020.00010.177

[CR44] Noroozian A, Amiri B, Kermani MAMA (2022) Investigation of cinematic genre diversity based on social network analysis: The lost ring of the Iranian cinema industry. Kybernetes. 10.1108/k-02-2022-0221

[CR45] Perretti F, Negro G (2007) Mixing genres and matching people: A study in innovation and team composition in hollywood. J Organiz Behav 28(5):563–586. 10.1002/job.464

[CR46] Pontikes E, Negro G, Rao H (2010) Stained red: A study of stigma by association to blacklisted artists during the “red scare”’ in Hollywood, 1945 to 1960. Am Sociolog Rev 75(3):456–478. 10.1177/0003122410368929

[CR47] Rossman G, Esparza N, Bonacich P (2010) I’d like to thank the Academy, team spillovers, and network centrality. Am Sociolog Rev 75(1):31–51. 10.1177/0003122409359164

[CR48] Rozenfeld HD, Song C, Makse HA (2010) Small-world to fractal transition in complex networks: A renormalization group approach. Phys Rev Lett 104(2):025701. 10.1103/physrevlett.104.025701 20366610 10.1103/PhysRevLett.104.025701

[CR49] Savk S, Dogu B (2021) Mapping yeşilçam: A relational approach to the Turkish film industry. CINEJ Cinema J 9(2):193–230. 10.5195/cinej.2021.425

[CR50] Senekal BA (2014) An investigation of Pierre de Wet’s role in the Afrikaans film industry using social network analysis (SNA). Literator 35(1):1099. 10.4102/lit.v35i1.1099

[CR51] Senekal BA, Stemmet JA (2014) The gods must be connected: An investigation of Jamie Uys’ connections in the Afrikaans film industry using social network analysis. Communication 40(1):1–19. 10.1080/02500167.2014.888361

[CR52] Shin S, Namgoong M, Park J (2022) Analysis of evolution of movies using massive movie-tag meme network data. J Korean Phys Soc 80(6):533–542. 10.1007/s40042-022-00454-6

[CR53] Spitz A, Horvát EÁ (2014) Measuring long-term impact based on network centrality: Unraveling cinematic citations. PLoS ONE 9(10):e108857. 10.1371/journal.pone.0108857 25295877 10.1371/journal.pone.0108857 PMC4189979

[CR54] Verhoeven D, Moore PS, Coles A, Coate B, Zemaityte V, Musial K, Prommer E, Mantsio M, Taylor S, Eltham B, Loist S, Davidson A (2020) Disciplinary itineraries and digital methods: Examining the kinomatics collaboration networks. NECSUS European Journal of Media Studies: 273–298. 10.25969/MEDIAREP/15320

[CR55] Verhoeven D, Musial K, Palmer S, Taylor S, Abidi S, Zemaityte V, Simpson L (2020) Controlling for openness in the male-dominated collaborative networks of the global film industry. PLOS ONE 15(6):e0234460. 10.1371/journal.pone.0234460 32530973 10.1371/journal.pone.0234460 PMC7292392

[CR56] Viana W, Santos PO, da Silva APC, Moro MM (2014) A network analysis on movie producing teams and their success. In: Proceedings of the 9th Latin American Web Congress, pp 68–76. IEEE

[CR57] Wasserman M, Mukherjee S, Scott K, Zeng XHT, Radicchi F, Amaral LAN (2014) Correlations between user voting data, budget, and box office for films in the internet movie database. J Associat Inform Sci Technol 66(4):858–868. 10.1002/asi.23213

[CR58] Watts DJ (1999) Networks, dynamics, and the small-world phenomenon. Am J Sociol 105(2):493–527. 10.1086/210318

[CR59] Xin X, Mossig I (2020) Governments and formal institutions shaping the networks of co-production in the Chinese and German film industries. Tijdschrift voor Economische en Sociale Geografie 112(3):220–238. 10.1111/tesg.12467

[CR60] Yahav I (2016) Network analysis: Understanding consumers’ choice in the film industry and predicting pre-released weekly box-office revenue. Appl Stoch Models Bus Ind 32(4):409–422. 10.1002/asmb.2156

[CR61] Zhang X, Wang J (2020) Transborder film production between mainland China and Hong Kong after CEPA: The interplay between political orientation and market forces. Tijdschrift voor Economische en Sociale Geografie 112(3):239–255. 10.1111/tesg.12466

[CR62] Zhang XJ, Tang Y, Xiong J, Wang WJ, Zhang YC (2019) How network topologies impact project alliance performance: Evidence from the movie industry. Entropy 21(9):859. 10.3390/e21090859

[CR63] Zhou Y (2022) Power, linkages and space: An exploratory study of China’s film cluster from 2010 to 2020. J Media Econom 34(3):176–193. 10.1080/08997764.2022.2115502

